# Orchard Net Covers Improve Resistance to Cherry Cracking Disorder

**DOI:** 10.3390/foods12030543

**Published:** 2023-01-26

**Authors:** Berta Gonçalves, Vânia Silva, Eunice Bacelar, Francisco Guedes, Carlos Ribeiro, Ana Paula Silva, Sandra Pereira

**Affiliations:** 1 Centre for the Research and Technology of Agro-Environmental and Biological Sciences (CITAB), Institute for Innovation, Capacity Building and Sustainability of Agri-Food Production, (Inov4Agro), University of Trás-os-Montes e Alto Douro (UTAD), Quinta de Prados, 5000-801 Vila Real, Portugal; 2 Cermouros-Cerejas de São Martinho de Mouros, Lda., Quinta da Ribeira, Bulhos, 4660-210 Resende, Portugal

**Keywords:** *Prunus avium* L., fruit cracking, net cover

## Abstract

Orchard net cover improves plant physiology, yield and fruit quality, pest and disease control, and anticipates fruit ripening. Moreover, this crop technology has been used to reduce natural cherry cracking (NCC). This is a serious physiological disorder that cracks the epidermis, the hypodermis, and the storage parenchyma layers of the fruit due to rainfall events near the harvest and it is related to low fruit osmotic potential and/or high fruit water permeability. This work aims to study the effect of orchard net cover on sweet cherry trees, cv. Early Bigi, in two harvesting years (2019 and 2021). The NCC, the induced cracking index (CI), and the cracking type incidence were determined. In addition, epicuticular and intra-cuticular wax content, biometric and physicochemical parameters were also evaluated. Net cover reduced the natural cracking index by 40%. High fruit weight values were observed in covered trees comparing to the control ones, with increases of 45% and 13%, in 2019 and 2021, respectively. A positive correlation was observed between CI and total soluble solids and a negative correlation between CI and wax content. Therefore, with forecasts of worsening heavy precipitation events near harvest, protecting cherry trees with nets will increase resistance to fruit cracking.

## 1. Introduction

Fruit cracking is a serious commercial problem in several types of fruit production worldwide, as it leads to huge losses in production and profits for producers. When used on sweet cherries, the term cracking is a generic term used to describe the rain-induced fracture of the fruit’s skin, sometimes associated with the rupture of the underlying flesh [1]. This physiological disorder has received special attention from researchers and most of them report that fruit cracking is caused by the excess uptake of water directly by the fruit surface, which results in localized breaks of the skin [2,3], or by the absorption of water through the tree vascular system [4]. According to other authors, fruit cracking can also be brought on by skin contracting as a result of a sudden drop in temperature or rapid cooling brought on by rain [5].

Rain-induced cherry cracking can be differentiated in macro- and micro-cracks. Macro-cracks can reach the epidermal and hypodermal cell layers and can be detected by visual inspection, while micro-cracks are not visible to the naked eye [3,6]. Fruit cracks can take three distinct forms: stem end cracks (SCR: stem cavity region), calyx end cracks (SSR: stylar scar region), and large cracks usually deep into the pulp on the cheek of the fruit (CR: cheek region) [7,8]. Usually, the stem cavity region and stylar scar region small cracks (base and top end of the fruit), which normally appear when cherries are not yet ripe, are accepted by consumers, if there is no fungal infection. On the contrary, fruits with cheek region cracks are generally refused by consumers [9].

The genetic susceptibility of cultivars and rootstocks, the environmental conditions, and the agronomic practices are the three main factors affecting sweet cherry cracking. Within the genetic susceptibility, both cultivars and rootstocks influence the fruit cracking disorder. In particular, the fruit size and shape, the firmness, the sugar content, and the exocarp properties can influence fruit cracking. In fact, previous studies show that larger fruits [8,10], firmer fruits [10], and/or fruits with lower intra- and epicuticular wax content [8] are more prone to cracking. Positive correlations were also observed, in previous studies, between sugar content and cracking index [11]. The rootstock and cultivar selection, and also the training system, influence the fruit cracking, since in compact trees, fruits are more protected against cracking due to rainfall [12].

The development of crack-resistant cultivars and the identification of genes involved in cracking resistance is a major objective in most breeding programs [13]. Lipid transfer protein (PaLTPG1), PaATT1, PaWINB, wax synthase (PaWS), and 3-ketoacyl-CoA synthase (PaKCS6) genes have been identified as being involved in the biosynthesis of cuticular wax and possibly with cracking development [14,15,16]. Genes encoding cell wall enzymes related to pectin metabolism and cell expansion (expansins) also play an important role in the cracking mechanism [13,16,17,18,19]. Furthermore, modifications in some metabolites content in sweet cherry cultivars, such as fucose and taxifolin (dihydroquercetin), in sweet cherry cultivars have been correlated with the cracking susceptibility [20].

The influence of the environmental conditions in the incidence of fruit cracking is related to the tension caused by water potential gradients between the soil, through the plant, and to the environment, which is the main factor responsible for water movement through plants [7]. In this sense, rainfall and high humidity during or near the harvest time increases the fruit cracking [21]. Furthermore, high temperature increases the water uptake and fruit transpiration, also increasing the fruit cracking as a consequence [10,21].

In relation to the agronomic practices, the foliar application of gibberellic acid, abscisic acid, salicylic acid, glycine betaine, calcium, and seaweed extracts (*Ascophyllum nodosum*) can influence fruit cracking [22], since these compounds can affect tree water relations. The use of soil covers can help to reduce cracking, because of lower water uptake by the roots [12]. Furthermore, irrigation and plant nutrition can also influence fruit cracking and, therefore, must be carefully managed.

The selection of appropriate cultivar, rootstock, and site is the most effective way, to minimize the rain damage in a cherry orchard. The use of late cultivars can be a strategy to reduce cracking in sites with high precipitation levels. It is also crucial to take in account the edaphoclimatic conditions of the site. If possible, little or no heavy rain incidence should be present in the selected site near harvest time.

The use of sprays of mineral salts, fungicides, and other chemicals for the reduction in fruit cracking has been extensively tested in recent years, and its effectiveness has been proven in several studies. Moreover, other agronomic practices, such as irrigation, fertilization and pruning can also influence fruit cracking.

Recently, the use of rain cover protection in cherry trees has gained importance as a strategy to reduce fruit cracking and fruit decay. These covers should be applied at the onset of fruit growth in stage III. Over the years, different covering systems have been produced and tested, from pole-and-wire tents to steel hoop houses or high tunnels. Although cracking reduction is the main objective of the covers, there are several additional benefits to each system, including not only practical management issues, but also significant improvements in crop physiology, fruit quality and productivity, pest/disease control, and early fruit maturity.

In this sense, the aim of this work is to compare the quality traits of fruits from sweet cherry trees (cv. Early Bigi), covered and uncovered, especially in relation to natural and induced cracking index and intra- and epicuticular wax content. Moreover, biometric attributes, chemical, chromatic, and texture parameters are performed. The choice of this cultivar was due to the fact that it is a very early cultivar, which allows the Resende region to anticipate the cherry harvest in relation to other regions of the country and even regions of Europe.

## 2. Materials and Methods

### 2.1. Experimental Trial

During 2019 and 2021, an experimental trial was carried out in a sweet cherry orchard installed in São João de Fontoura, Resende (41°12′ N, 7°93′ W, 149 m above sea level). In the year 2020, it was not possible to carry out all the assays presented in this article, due to the restrictions of the COVID-19 pandemic, therefore, the results of that year are not presented.

The meteorological data were collected by a station placed close to the orchard, and the temperatures and rainfall between January and May of each year are shown in Table 1.

For this study, 10 covered and 10 uncovered cv. Early Bigi cherry trees grafted onto Santa Lucia 64 rootstock and with 10 years old trained vertical axis were randomly selected. The distance between trees was 3.0 m between rows and 2.5 m within the row. Trees were covered with a white polyethylene net, with 2,1 mm, and 250 cm monofilament knitted-net, rainproof (Gore-Tex effect) and anti-insect. Fruits were collected at the commercial ripening stage. From the 10 trees under study in each situation, around 500 g of fruit samples were randomly collected from each group of trees. Additionally, 2 fruits per tree were also collected to determine the intra and epicuticular wax content. Fruits were randomly separated into 2 groups, the first consisting of 30 sweet cherries destined to determine the fruit weight and size, chromatic parameters, epidermis rupture force and flesh firmness, total soluble solids, titratable acidity, maturity index, and pH measurements, while the second (150 healthy cherry fruits) was used for the induced cracking index evaluation.

### 2.2. Natural Cracking Index

In the field, during the harvest, the natural cracking index was evaluated. In this sense, the healthy and cracked or rotten fruits of each tree under study were counted (by weighing).

### 2.3. Induced Cracking Index and Crack Type Incidence

The induced cracking index was evaluated in the second group of fruits according to the following adapted procedure [23,24]. Thus, 50 cherries were immersed into 3 containers filled with 2 L of distilled water each. After 2, 4, and 6 h, the presence of macroscopic cracks in the cherries was checked. At each time, the cracked fruits were removed from the container, and cherries without cracks were kept in the bath. Then, the cracking index was calculated:

CI = ((5a + 3b + c) × 100)/250

a, b, and c represent the cracked fruit number at 2, 4, and 6 h of water immersion, respectively.

The crack type incidence (%) was also calculated by the ratio between the number of cracks of a particular type (SCR, SSR, and CR) and the total number of cracks.

### 2.4. Epicuticular and Intra-Cuticular Wax Content

The epicuticular and intra-cuticular wax content of sweet cherries was evaluated according to Hamilton [25]. For this procedure, 2 fruits (per tree) without peduncle and previously weighed were immersed for 2 min in a mixture of chloroform–methanol (3:1). Then, the cherry fruits were rejected and the solution was filtered into a glass, properly identified and previously weighed. The glasses were left to dry, and then they were weighed again. Thus, the fruit weight and the difference in the weights were used to determine the wax content (μg g^−1^ of fresh weight).

### 2.5. Quality Attributes of the Fruits

#### 2.5.1. Weight and Dimensions

Biometric attributes of the fruits were evaluated using the thirty sweet cherries of the first group. The cherry weight (g) was determined with an electronic balance (EW2200-2NM, Kern, Balingen, Germany) and the size was evaluated by determining the width and the largest and smallest diameters (mm), with an electronic caliper (500-196-30, Mitutoyo, Andover, UK).

#### 2.5.2. Chromatic Parameters

To evaluate the color of the fruit, a measurement was made on both sides of these same 30 fruits with a colorimeter (CR-300, Minolta, Osaka, Japan), in accordance with the Commission International de l’Eclairage (CIE) system of 1976, using the three-dimensional space (CIELAB). In this system, color was described either by lightness (L*) read from 0 (‘opaque’ or ‘black’) to 100 (‘transparent’ or ‘white’), coordinate a* (green to red content), and coordinate b* (blue to yellow content), or by the use of cylindrical brightness coordinates (L*), hue (*h*°), and chroma (C*), directly related to the Munsell coordinates [26,27]. The hue angle (°), hue = arctg (b*/a*), expressed the color nuance [26], and values were defined as follows: red–purple: 0°, yellow: 90°, bluish–green: 180°, and blue: 270° [28]. The chroma (C*) of cherry fruits was expressed according to the formula: C* = (a*^2^ + b*^2^)^1/2^ [29,30,31].

#### 2.5.3. Epidermis Rupture Force—ERF and Flesh Firmness—FF

Thirty sweet cherries from the first group were also used to determine ERF (N) and FF (N mm^−1^), through a TA.XT.plus texture analyser (Stable Micro Systems, Godalming, UK), applying a 30 N loading cell and a 2.0 mm diameter cylindrical probe (P2). The maximum force to a compression of 5 mm was determined at a speed of 1 mm s^−1^.

#### 2.5.4. Total Soluble Solids, Titratable Acidity, Maturity Index, and pH

Immediately after texture determination, cherries were separated into 3 batches of ten fruits each. In the juice obtained, using an electric extractor (ZN350C70, Tefal Elea, Hong Kong, China), the total soluble solids (°Brix)—TSS—were evaluated, with a digital refractometer (PR-101, Atago, Tokyo, Japan) and expressed as a percentage of soluble solids in the juice (%). The pH was also determined using a pH meter (3310 Jenway). Titratable acidity (g malic acid 100 g^−1^ of fresh weight)—TA—was evaluated in a solution of 10 mL of fruit juice and 10 mL of distilled water, through a titration with sodium hydroxide (NaOH) (0.1 mol L^−1^) until pH 8.2, using a Schott Easy Titroline automatic titrator. The maturity index was calculated by the ratio between TSS and TA.

### 2.6. Statistical Analysis

The analysis of the results was performed in the Software SPSS V.25 (SPSS-IBM, Corp., Armonk, NY, USA) using one-way analysis of variance (ANOVA), followed by Tukey’s post hoc multiple range test (*p* < 0.05). The fulfillment of the ANOVA requirements, namely, the normal distribution of the residuals and the homogeneity of variance, were evaluated using the Shapiro–Wilk’s test (*n* < 50), and the Levene’s test, respectively. All dependent variables were analyzed using a one-way ANOVA with or without Welch correction, depending on if the requirement of the homogeneity of variances was fulfilled.

A principal component analysis (PCA) was also performed. PCA was performed by eigenvalue decomposition of the data correlation (corr-PCA) matrix after normalizing the data matrix for each variable.

## 3. Results and Discussion

### 3.1. Effect of Net Cover on Fruit Cracking Parameters

#### 3.1.1. Natural Cracking Index

**Figure 1:**
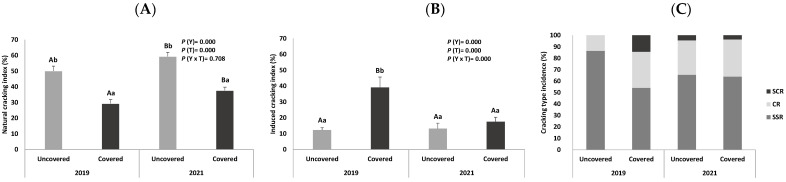
(**A**) Natural cracking index (%), (**B**) induced cracking index (%), and (**C**) incidence of the different types of cracking in sweet cherry fruits (cv. Early Bigi) uncovered and covered during 2 harvesting years (2019 and 2021). Data are expressed as mean ± standard deviation. Different capital letters correspond to significant differences (*p* < 0.05) between years within each treatment; different small letters correspond to significant differences (*p* < 0.05) between treatment for the same year. Types of cracking: SCR: stem cavity region, CR: cheek region, and SSR: stylar scar region.

The percentages of healthy and cracked sweet cherry fruits from uncovered and covered trees, collected in two different harvesting years (2019 and 2021), are presented in Figure 1A. The percentage of cracked fruits is higher in uncovered trees (50.09 and 59.35%, for 2019 and 2021, respectively) compared to the covered trees (29.38 and 37.66 for each year, respectively), enhancing the protective role of coverings against cracking, as demonstrated in previous studies [32,33,34].

#### 3.1.2. Induced Cracking Index and Crack Type Incidence

The induced CI and the crack type incidence of the sweet cherry fruits from uncovered and covered trees, collected in two different harvesting years (2019 and 2021) are presented in Figure 1B,C, respectively.

It is observed that cherries from covered trees in 2019 present a very high percentage of induced cracking in relation to fruits from uncovered trees harvested in the same year, as well as in relation to fruits from covered trees harvested in the year 2021, probably due to the low content of cuticular waxes observed in these trees.

Regarding the crack type incidence, the predominant type of cracking in all fruits evaluated is stylar scar region (SSR) cracking, followed by cheek region (CR) cracking. To a lesser extent, stem cavity region (SCR) cracking is observed in fruits collected in 2021 (from covered and uncovered trees), as well as in fruits from covered trees in 2019. The higher SSR cracking incidence can be the result of the higher osmotic concentration in this part of the fruit. It accounts for faster water absorption by the skin, leading to a quicker crack formation [24]. According to Christensen, the small SCR and SSR cracks usually occur in a state of very early maturation. The same author [9] states that fruits with these cracks are well-accepted by consumers. However, Pereira et al. [8] state that fruits with CR cracks are usually rejected.

#### 3.1.3. Epicuticular and Intra-Cuticular Wax Content

**Figure 2:**
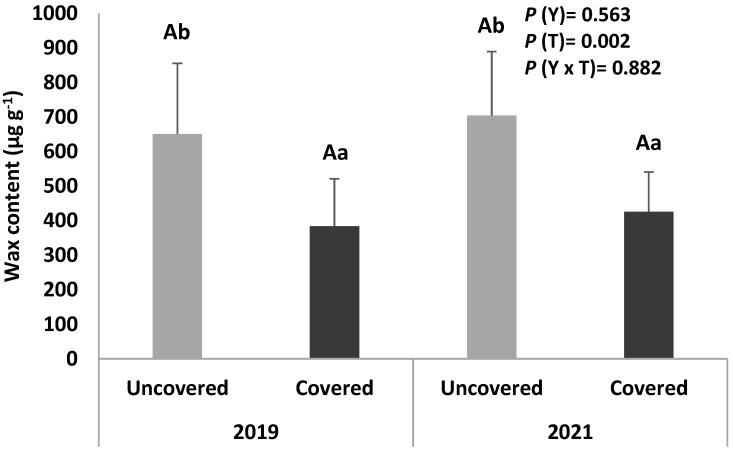
Epicuticular and intra-cuticular wax content of sweet cherry fruits (cv. Early Bigi) uncovered and covered, during 2 harvesting years (2019 and 2021). Data are expressed as mean ± standard deviation. Different capital letters correspond to significant differences (*p* < 0.05) between years within each treatment; different small letters correspond to significant differences (*p* < 0.05) between treatment for the same year.

The epicuticular and intra-cuticular wax content of the sweet cherry fruits from uncovered and covered trees collected in two different harvesting years (2019 and 2021) is presented in Figure 2.

Regarding the effect of the coverings, it is verified that in both years, the cuticular wax content is higher in the fruits from uncovered trees, which may have led to the lower induced cracking index observed in these fruits. In addition, there are no significant differences between the two years evaluated.

### 3.2. Effect of Cover Net on Other Quality Attributes of the Fruits

#### 3.2.1. Weight and Dimensions

The biometric attributes of the sweet cherry fruits from uncovered and covered trees collected in two different harvesting years (2019 and 2021) are presented in Table 2. Fruit size is one of the main factors responsible for consumer preference and market price.

In both years, despite the great variability in meteorological conditions, the orchard cover increases the caliber of the fruits. Comparing the two years evaluated, there are significant differences in cherries from uncovered trees, with the year 2021 presenting the largest and heaviest fruits, probably due to the greater availability of water in the soil, due to the greater precipitation observed in that year. Indeed, it has been suggested that changes in fruit quality, in particular fruit caliber, under protective netting are often more influenced by the environmental conditions in that specific growing season than by the netting itself [35].

#### 3.2.2. Chromatic Parameters

The chromatic parameters of sweet cherry fruits from uncovered and covered trees collected in two different harvesting years (2019 and 2021) are presented in Table 3. Fruit color is another important quality trait [36], since it also directly influences consumer acceptance.

In 2019, the highest chromatic values are observed in cherries from uncovered trees, thus, indicating less mature cherries than those from covered trees. In fact, fruits from covered trees present darker fruits (L* = 48.72) than fruits from uncovered trees (L* = 52.90). The other chromatic parameters follow the same trend, corroborating the results obtained in other studies, which state that the chroma and the hue angle of less ripe cherries are always higher than in the ripe ones [37].

On the other hand, in 2021, the results are contrary, since, in general, the chromatic parameters are higher in cherries from covered trees, indicating that the coverings delayed fruit maturation this year. This can be explained by the higher maximum temperatures observed in March and April 2021 compared to 2019, which led to a greater maturation of the fruit from the uncovered trees.

Comparing the years, the data are variable, being that the cherries harvested in 2019 show higher values of b* and hue angle, and lower values of a*.

#### 3.2.3. Epidermis Rupture Force and Flesh Firmness

The texture parameters (epidermis rupture force: ERF and flesh firmness: FF) of the sweet cherry fruits from uncovered and covered trees collected in two different harvesting years (2019 and 2021) are presented in Table 4.

Fruit firmness is an important trait in these fruits, since it is related to a better resistance to deterioration and mechanical damage, leading to an increase in the shelf-life period [38].

Cherries from uncovered trees show, in general, significantly higher values of ERF and FF in both years, with exception of the FF in 2019, in which no significant differences are observed.

Comparing the two years evaluated, it is found that the ERF is significantly higher in 2019 in cherries from trees with coverage. In relation to FF, this parameter is higher in 2021 for both fruits from uncovered and covered trees.

According to Wang and Long [39], fruits with higher flesh firmness present fewer physiological disorders during handling, storage, and shipping.

#### 3.2.4. Total Soluble Solids, Titratable Acidity, Maturity Index, and pH

The chemical parameters of the sweet cherry fruits from uncovered and covered trees, collected in two different harvesting years (2019 and 2021) are presented in Table 5.

In 2019, fruits from covered sweet cherry trees present significantly higher TSS and pH values compared to cherries from uncovered trees. On the other hand, in 2021, significant differences in the TSS and maturity index are observed in fruits from uncovered trees, thus, showing a contrary trend to the year 2019.

Comparing between years, the pH is significantly higher in 2021, for all analyzed systems.

Cherry ripening is associated with changes in color, sugar, vitamin, and organic acid [40]. The sugars in sweet cherry fruits are mainly glucose and fructose [41], with lower amounts of sorbitol and very low quantities of sucrose [42]. The obtained TSS values (between 8.27 and 11.22 °Brix) are considered very low, considering the reference values for sweet cherries. For example, Crisosto et al. [43] suggested a TSS value above 14 °Brix for sweet cherries to be acceptable for marketing. However, considering the precocity of the Resende region compared to other sweet cherry regions in Portugal, and even throughout Europe, these first fruits are very profitable for the producer, and even without the characteristic sweetness of later cherries, they are well accepted by the consumers.

The TA follows the same profile of TSS, being higher in fruits from covered trees in 2019 and in fruits from uncovered trees in 2021. Indeed, TSS content is positively correlated with titratable acidity (R^2^ = 0.691, *p* = 0.003). In sweet cherry, TA is mainly (97.6%) due to malic acid and, to a lesser extent, to citric (1.9%) and quinic acids (0.5%) [44]. The obtained values (between 0.47 and 0.60 g of malic acid per 100 g^−1^) are higher than those obtained in other studies [45], which is normal since Early Bigi is an early cultivar and, therefore, generally more acidic.

Sweetness can be expressed as total soluble solids and sourness as titratable acidity, with the TSS/TA ratio considered as overall taste attribute: maturity index [46,47]. The optimal ratio varies from 12.30 to 23.50 [48]. The obtained values (between 17.30 and 19.10) are normal for earlier cultivars, such as Early Bigi, considering the low TSS content and the high TA.

In 2019, the fruits from covered trees present a higher maturity index and simultaneously a darker color, also indicating riper fruits. Contrariwise, in 2021, the maturity index is lower in fruits from covered trees, which, at the same time, present a lighter color.

### 3.3. Correlations

A positive correlation is observed between cracking index and TSS (R^2^ = 0.658, *p* = 0.006), indicating that ripe fruits are more prone to splitting. However, negative correlations are observed between cracking index and wax content (R^2^ = −0.608, *p* = 0.012). In general, the lower the wax content, the greater the water intake for the fruit, and, consequently, the higher the cracking index [8]. According to Yamaguchi et al. [49], cultivars more tolerant to cracking have longer periods of cell division, resulting in a larger fruit mesocarp and higher wax content.

Comparing the natural and the induced cracking index, an opposite trend is observed, showing that cherries from covered trees tend to split, and this does not happen, thanks to the protective role of the covers.

Similar to the texture parameters, the wax content is also lower in fruits from covered trees. In fact, a positive correlation is observed between the wax content and the ERF (R^2^ = 0.751, *p* = 0.001).

On the other hand, negative correlations are observed between the wax content and all biometric attributes (R^2^ = −0.530, *p* = 0.035; R^2^ = −0.559, *p* = 0.025; and R^2^ = −0.621, *p* = 0.010 for weight, larger diameter, and height of sweet cherries, respectively), suggesting that the wax content is lower in larger fruits.

The ERF presents negative correlations with all the biometric attributes (R^2^ = −0.844, *p* = 0.000; R^2^ = −0.741, *p* = 0.001; R^2^ = −0.883, *p* < 0.001, and R^2^ = −0.753, *p* = 0.001 for weight, height, larger, and smaller diameter of fruits, respectively). This indicates that bigger fruits require a lower epidermis rupture force, and, consequently, are more likely to crack. Indeed, the ERF and the cracking index are negatively correlated in this work (R^2^ = −0.561, *p* = 0.024).

### 3.4. Principal Component Analysis

**Figure 3:**
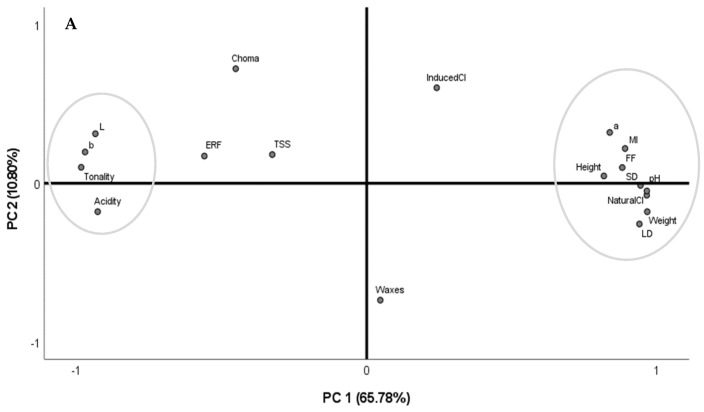

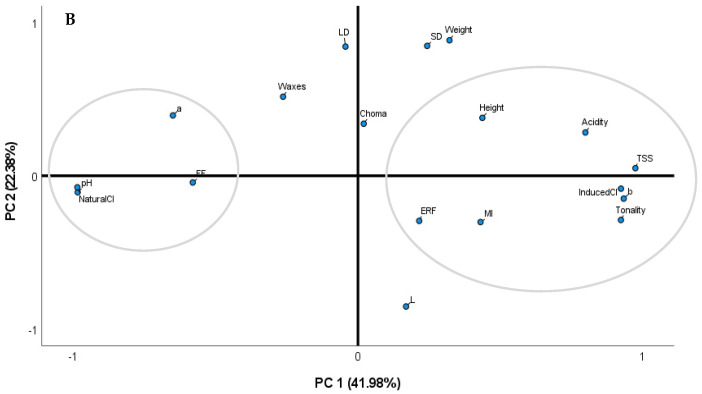
Principal component analysis using the whole dataset of fruits from sweet cherry trees (cv. Early Bigi) uncovered (**A**) and covered (**B**), during 2 harvesting years (2019 and 2021).

To better understand the correlations between all the evaluated parameters, chemometric analyses were performed integrating all the data (Figure 3A,B). The PCA based on the correlation matrix standardizes the data, and this analysis was performed using a correlation matrix (corr-PCA). In uncovered trees, the PC1 and PC2 represent 76.58% of the variance, with factor 1 showing the higher weight (65.78%). In covered trees, these factorial axes (PC1 and PC2) represent 64.36% of the total variance, with factor 1 presenting 41.98%.

Considering the performed PCA with uncovered trees, two groups perfectly separated in space can be observed. The first group comprises the chromatic parameters L*, b*, and tonality and the acidity, while the second group includes all the biometric parameters, pH, maturity index, flesh firmness, a*, and natural cracking index. Although the natural and the induced cracking index are not too close, these two parameters are in the same quadrant.

Considering the PCA performed with covered trees, the biometric parameters, chemical parameters (TSS, acidity, and MI), the tonality, the ERF, and the induced cracking index are in one group, while the pH, the FF, the chromatic parameter a*, and the natural cracking index are in another group, showing the contrary tendencies of the natural and the induced cracking index in these trees.

## Figures and Tables

**Table 1:** 2019 and 2021.

Month	Parameter	2019	2021
January	Minimum temperature (°C)	−0.9 to 9.4	−2.3 to 12.4
	Maximum temperature (°C)	5.1 to 16.2	3.9 to 17.4
	Total rainfall (mm)	5.4	152.2
February	Minimum temperature (°C)	−0.5 to 9.1	1.7 to 11.4
	Maximum temperature (°C)	9.2 to 22.4	10.1 to 20.8
	Total rainfall (mm)	3.5	164
March	Minimum temperature (°C)	3.7 to 12.2	2.8 to 14.3
	Maximum temperature (°C)	11.9 to 22.6	13.9 to 27.7
	Total rainfall (mm)	4.1	4.8
April	Minimum temperature (°C)	2.0 to 14.3	4.8 to 13.7
	Maximum temperature (°C)	10.9 to 26.0	14.0 to 23.8
	Total rainfall (mm)	8.1	90.4
May	Minimum temperature (°C)	6.4 to 17.4	5.0 to 14.9
	Maximum temperature (°C)	16.2 to 32.9	14.9 to 29.7
	Total rainfall (mm)	0.3	49.2

**Table 2:** Harvesting years (2019 and 2021). Different capital letters correspond to significant differences (*p* < 0.05) between years within each treatment; different small letters correspond to significant differences (*p* < 0.05) between treatment for the same year.

Year	Treatment	Weight	Height	Larger Diameter (mm)	Smaller Diameter (mm)
(Y)	(T)	(g)	(mm)		
2019	Uncovered	6.10 ± 0.88 Aa	21.26 ± 0.96 Aa	24.46 ± 1.26 Aa	19.59 ± 1.09 Aa
	Covered	8.83 ± 1.21 Ab	23.27 ± 1.10 Ab	27.04 ± 1.57 Ab	21.76 ± 1.67 Ab
	*p*-value	0.000	0.000	0.000	0.000
2021	Uncovered	7.56 ± 1.51 Ba	21.77 ± 1.37 Aa	25.57 ± 1.96 Ba	21.08 ± 1.82 Ba
	Covered	8.53 ± 1.18 Ab	22.78 ± 1.43 Ab	27.09 ± 1.29 Ab	21.39 ± 1.32 Aa
	*p*-value	0.007	0.007	0.001	0.441
	*P* (Y)	0.009	0.956	0.023	0.046
	*P* (T)	0.000	0.000	0.000	0.001
	*P* (Y × T)	0.001	0.071	0.036	0.005

**Table 3:** Harvesting years (2019 and 2021). Different capital letters correspond to significant differences (*p* < 0.05) between years within each treatment; different small letters correspond to significant differences (*p* < 0.05) between treatment for the same year.

Year	Treatment	Chromatic Parameters				
(Y)	(T)	L*	a*	b*	Chroma (C*)	Hue Angle (°)
2019	Uncovered	52.90 ± 5.17 Bb	34.47 ± 3.93 Aa	25.57 ± 1.69 Bb	42.98 ± 3.49 Ab	36.75 ± 3.43 Bb
	Covered	48.72 ± 4.96 Aa	34.13 ± 2.59 Aa	23.26 ± 2.30 Ba	41.34 ± 2.96 Aa	34.26 ± 2.61 Ba
	*p*-value	0.000	0.581	0.000	0.006	0.000
2021	Uncovered	43.50 ± 7.31 Aa	38.28 ± 5.01 Bb	17.77 ± 2.78 Aa	42.25 ± 5.33 Aa	24.91 ± 3.11 Aa
	Covered	49.41 ± 7.50 Ab	36.23 ± 4.89 Ba	18.85 ± 2.58 Ab	40.98 ± 4.34 Aa	27.73 ± 5.09 Ab
	*p*-value	0.000	0.025	0.030	0.154	0.000
	*P* (Y)	0.002	0.004	0.000	0.374	0.000
	*P* (T)	0.485	0.345	0.346	0.192	0.953
	*P* (Y × T)	0.002	0.457	0.019	0.693	0.023

**Table 4:** Harvesting years (2019 and 2021). Different capital letters correspond to significant differences (*p* < 0.05) between years within each treatment; different small letters correspond to significant differences (*p* < 0.05) between treatment for the same year.

Year (Y)	Treatment (T)	Epidermis Rupture Force (N)	Flesh Firmness (N mm^−1^)
2019	Uncovered	3.74 ± 0.66 Ab	0.87 ± 0.17 Aa
	Covered	2.91 ± 0.53 Ba	0.93 ± 0.18 Aa
	*p*-value	0.000	0.154
2021	Uncovered	3.54 ± 0.57 Ab	1.33 ± 0.35 Bb
	Covered	2.85 ± 0.71 Aa	1.08 ± 0.38 Ba
	*p*-value	0.000	0.008
	*P* (Y)	0.214	0.000
	*P* (T)	0.000	0.144
	*P* (Y × T)	0.498	0.015

**Table 5:** Harvesting years (2019 and 2021). Different capital letters correspond to significant differences (*p* < 0.05) between years within each treatment; different small letters correspond to significant differences (*p* < 0.05) between treatment for the same year.

Year (Y)	Treatment (T)	Total Soluble Solids (°Brix)	pH	Titratable Acidity (g Malic Acid 100 g^−1^)	Maturity Index
2019	Uncovered	9.80 ± 0.20 Aa	3.62 ± 0.02 Aa	0.57 ± 0.04 Aa	17.28 ± 1.43 Aa
	Covered	11.22 ± 0.42 Bb	3.79 ± 0.04 Ab	0.60 ± 0.10 Aa	19.09 ± 2.67 Aa
	*p*-value	0.001	0.001	0.624	0.359
2021	Uncovered	9.63 ± 0.35 Ab	4.06 ± 0.05 Ba	0.50 ± 0.02 Aa	19.14 ± 0.55 Ab
	Covered	8.27 ± 0.55 Aa	4.14 ± 0.01 Ba	0.47 ± 0.02 Aa	17.76 ± 0.57 Aa
	*p*-value	0.022	0.067	0.070	0.039
	*P* (Y)	0.000	0.000	0.001	0.683
	*P* (T)	0.862	0.000	0.957	0.742
	*P* (Y × T)	0.000	0.003	0.165	0.028

## Data Availability

The data are avaliable from the corresponding author.
